# Correlation of Amine Concentrations in Blood and Cerebrospinal Fluid in Healthy Volunteers and Migraineurs

**DOI:** 10.3390/ijms26209899

**Published:** 2025-10-11

**Authors:** Aster V. E. Harder, Jan B. van Klinken, Robin M. van Dongen, Gerrit L. J. Onderwater, Michel D. Ferrari, Amy C. Harms, Thomas Hankemeier, Gisela M. Terwindt, Arn M. J. M. van den Maagdenberg

**Affiliations:** 1 Department of Neurology, Leiden University Medical Centre, 2333 ZA Leiden, The Netherlands; a.v.e.harder@lumc.nl (A.V.E.H.);; 2 Department of Human Genetics, Leiden University Medical Centre, 2333 ZC Leiden, The Netherlands; 3 Department of Clinical Chemistry, Laboratory Genetic Metabolic Disease, Amsterdam University Medical Centre, 1105 AZ Amsterdam, The Netherlands; 4 Division of Analytical Biosciences, Leiden Academic Centre for Drug Research, 2333 AL Leiden, The Netherlands

**Keywords:** omics, neurotransmitters, amines, migraine, pain, cerebrospinal fluid, blood, UPLC-MS

## Abstract

Many central nervous system disorders (CNS), including chronic pain and migraine, involve metabolic changes in the brain. These changes are best detected and monitored in cerebrospinal fluid (CSF), which requires lumbar puncture. Blood-based measurements may offer an alternative, if they reflect CSF changes. To assess this, we measured and correlated the concentrations of 39 amino acids, biogenic amines, and other amines in blood and CSF of 95 healthy volunteers and, in addition, correlated the ratios of 741 amines. Amines were measured using a validated UPLC-MS platform. In healthy volunteers, only 4/39 (10.3%) analyzed amine metabolite concentrations had a correlation coefficient ≥ 0.70. Correlations of metabolite ratios were significantly better for 308/741 (41.5%) combinations. Specifically, ratios of amino acids showed high correlations. In addition, amines were investigated in 197 participants with migraine. Six amine metabolite ratios were different in migraineurs versus healthy volunteers. Most blood amine concentrations do not reflect those in CSF, but many of the ratios did correlate between CSF and plasma, showing diagnostic potential. This study improves our understanding of blood-CSF relationships, and our data suggest that ratios of amines may be of relevance to CNS disorders, as we showed for migraine.

## 1. Introduction

Chronic pain conditions constitute among the most profound causes of disability worldwide and are an enormous burden for the individuals affected and the society at large [1,2]. Various chronic pain conditions are known to be associated with alterations in cerebrospinal fluid (CSF) metabolites [3,4]. Amines, such as neurotransmitters and their precursors like glutamate, γ-aminobutyric acid (GABA), glutamine, serotonin, and tryptophan, are thought to be involved in the pathophysiology of not only chronic pain but also of diseases such as migraine, which is characterized by painful attacks [5,6,7]. To detect and monitor metabolic changes in the brain, metabolites can be measured in the CSF. Novel metabolomics techniques can reliably quantify an increasing number of metabolites in CSF and other body fluids [8,9]. CSF sampling, however, requires lumbar punction, which has ethical concerns and has been linked to adverse events, such as post-dural headache [10]. The risk of such headache can be reduced, but not completely negated, using non-traumatic lumbar needles [11,12]. For assessing dynamic changes in the brain—for example, in the case of a paroxysmal brain disorder such as migraine where you would like to compare changes outside and during an attack—repeated CSF sampling would be optimal. This, however, is neither ethical nor realistic. Measuring metabolites in repeated blood samples would be a logical and less burdensome alternative for patients, provided that metabolic changes in blood reliably reflect those in the CSF. Although blood samples are often used in biomarker research for diagnosis and/or disease progression studies for many CNS diseases, it is often not clear whether and to what extent blood samples are representative of what happens in the brain. Given the important role of amines in the brain, it is highly relevant to know whether amines measured in blood and CSF samples correlate. Studies in neurodegenerative disease often focus on both blood and CSF measurements, considering specific metabolites or broader metabolomics; they do not compare the levels directly or via ratios. Rarely, they indirectly compare by looking at whether age has a similar effect on metabolites in both CSF and blood [13].

Older studies that did look at correlations between CSF and blood suggest that the levels of some amines in blood correlate well with those in CSF [14,15]. For some amines, there was a correlation only for CSF/blood ratios, possibly because these amines use the same transporter system across the blood–brain barrier [15]. Correlation was particularly prominent for five neutral amino acids: isoleucine, leucine, phenylalanine, valine, and tyrosine [15]. All in all, this may mean that correlations between blood and CSF levels are not so much about the concentrations themselves, but rather the ratios, which offer better opportunities to investigate metabolite changes in CNS diseases.

In the current study, we investigated, initially in heathy volunteers, whether and to what extent amine levels and amine ratios in blood correlate with those in CSF by using our validated amine metabolomics platform for CSF and blood [16]. While older studies mainly focused on proteinogenic amino acids, our platform allows for the determination of a much broader range of amines with much higher precision. Next, to apply our methodology in a pain disorder and perhaps gain further insight in its pathophysiology, we investigated the correlation of amine levels and/or ratios between CSF and blood in participants with migraine outside of attacks (interictally). Migraine is characterized by recurrent attacks of severe headache and other neurological symptoms. The two main subtypes of migraine are migraine with aura and migraine without aura, depending on whether patients also suffer from an aura, which is characterized by temporary disturbances that affect vision and sometimes also manifest as a tingling feeling in an arm or even a speech disturbance, before the headache [17].

## 2. Results

### 2.1. Amines in Plasma and CSF of Healthy Volunteers

#### 2.1.1. Concentrations of Amines

Forty overlapping amines in both CSF and plasma were measured in healthy volunteers after the first quality control (QC) steps. Additional QC demonstrated that in CSF, *O*-phosphoethanolamine showed abnormally high concentrations (>3 SDs above median) in multiple samples from one batch and was therefore excluded, leaving 39 amines for further analysis. No other metabolite in plasma nor CSF showed such batch effect. At the individual level, principal component analysis (PCA) identified one clear outlier in both plasma and CSF that was therefore excluded, leaving amine profiles of 95 healthy volunteers for further analysis (Table 1). Later, it turned out that the excluded participant suffered from cardiac disease. In healthy volunteers, most amine concentrations were higher in plasma than in CSF (Table 2). Only concentrations of ethanolamine and putrescine were higher in CSF than in plasma. L-Glutamine was the most abundant amine in CSF (median: 591 mM, interquartile range: 539–638 mM), with a similar concentration in blood (median: 665 mM, interquartile range: 600–715 mM).

#### 2.1.2. Correlations Between Single-Metabolite Levels in Plasma and CSF in Healthy Volunteers

**Figure 1:**
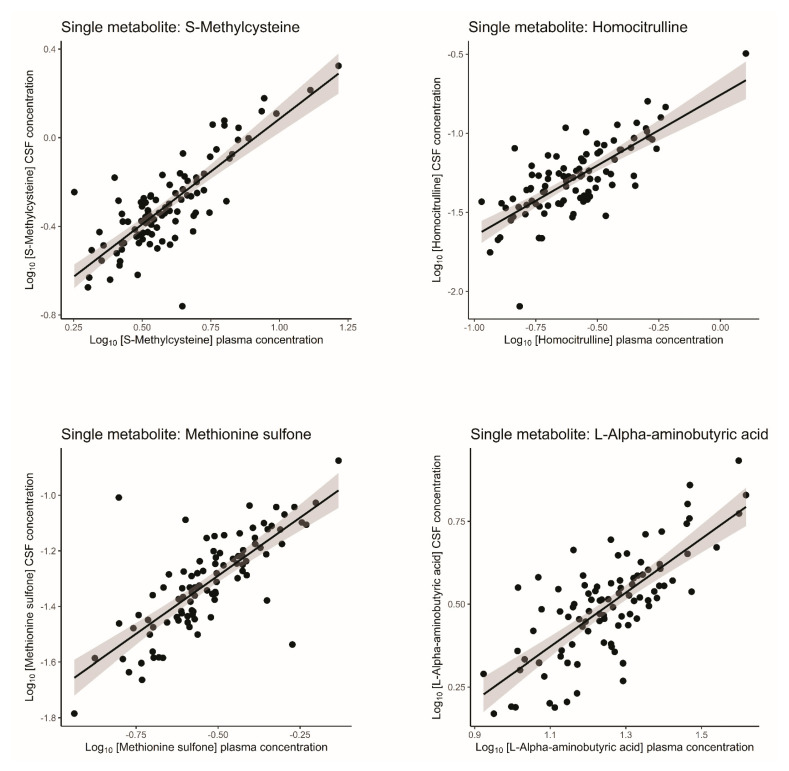
Correlation plots of amines with strongest correlation between plasma and CSF in healthy volunteers. Log_10_-transformed concentrations are plotted with plasma concentrations on the *X*-axis and CSF concentrations on the *Y*-axis. Values below [mean − 4 * SD] and above [mean + 4 * SD] were replaced with these cut-offs. Plots are based on uncorrected data. The black line indicates the linear regression line and the grey area indicates the 95% confidence interval. CSF = cerebrospinal fluid.

Correlation coefficients (*r*) and coefficients of determination (R^2^) were calculated to indicate the percentage of variation explained by a linear model. Most amine concentrations showed a low correlation between plasma and CSF ($r=\frac{{Amine\;x}_{\;plasma}}{{Amine\;x}_{\;CSF}}$) (Table 3). A total of 4 of the 39 amines (10.3%) had a correlation coefficient ≥ 0.70, namely, homocitrulline, S-methylcysteine, methionine sulfone, and L-alpha-aminobutyric acid (Table 3). Figure 1 shows the correlation plots of the log_10_-transformed uncorrected data of the four best correlating amines; the remaining plots can be found in the Supplementary Data (Supplementary Figure S1). The four amines showed no obvious sex differences (Table S1). Moreover, L-Threonine showed a higher correlation in females, SDMA showed a lower concentration in females compared to males, L-Aspargine and L-Glutamine showed lower concentration in males, and Gamma-Glutamylglutamine showed a higher concentration in males compared to females.

#### 2.1.3. Correlations Between Amine Metabolite Ratios in Plasma and CSF in Healthy Volunteers

**Figure 2:**
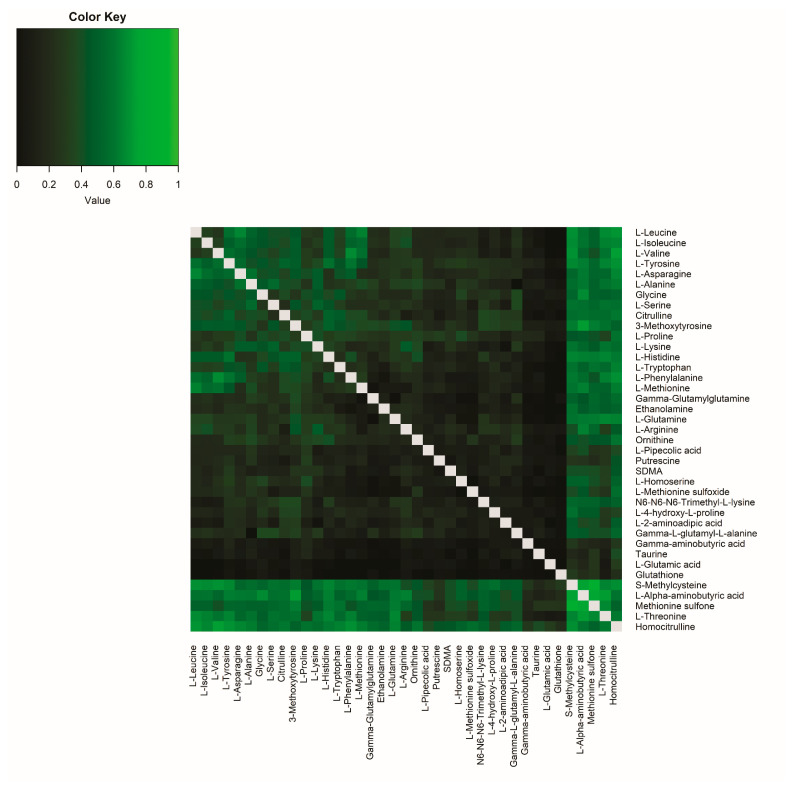
Correlation matrix of all possible metabolite ratios. Coefficients of determination (R^2^) of all possible metabolite ratios are plotted in healthy volunteers. The higher the R^2^, the brighter the square. Grey squares: ratio not applicable.

**Figure 3:**
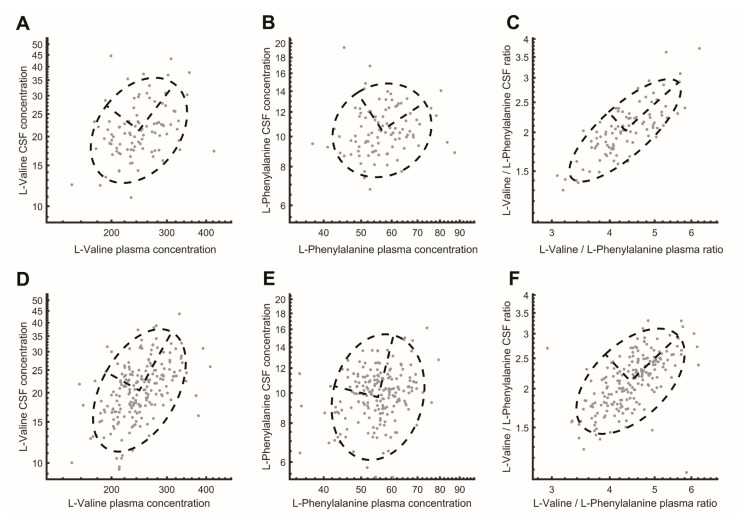
Example of single-metabolite vs. metabolite ratio correlation. Plasma concentrations are plotted on the *X*-axis and CSF concentrations on the *Y*-axis. Values below [mean − 4 * SD] and above [mean + 4 * SD] were replaced with these cut-offs. Plots are based on uncorrected data. (**A**) Correlation between plasma and CSF concentrations of L-Valine in healthy volunteers. (**B**) Correlation between plasma and CSF concentrations of L-Phenylalanine in healthy volunteers. (**C**) Correlation between plasma ratio of L-Valine/L-Phenylalanine and CSF ratio of L-Valine/L-Phenylalanine in healthy volunteers. (**D**) Correlation between plasma and CSF concentrations of L-Valine in participants with migraine. (**E**) Correlation between plasma and CSF concentrations of L-Phenylalanine in participants with migraine. (**F**) Correlation between plasma ratio of L-Valine/L-Phenylalanine and CSF ratio of L-Valine/L-Phenylalanine in participants with migraine. The dashed circle represents the 95% confidence interval of the Gaussian distribution. CSF = cerebrospinal fluid.

Next, we tested whether metabolite ratios in plasma correlated with the same ratio in CSF. Correlations between all possible ratios were calculated based on their R^2^ (Figure 2, Table S2). As expected, metabolites that already had strong single correlations (i.e., homocitrulline, S-methylcysteine, methionine sulfone, L-alpha-aminobutyric acid, and L-threonine) also showed strong correlation for most ratios with other amines. In addition, several other metabolite pairs showed high correlation. For example, for the single L-Valine correlation the R^2^ was 0.11 and for the single L-Phenylalanine correlation the R^2^ was 0.01, but the plasma ratio of L-Valine/L-Phenylalanine had a higher correlation with the CSF ratio of L-Valine/L-Phenylalanine ($\frac{{L-Valine}_{\;plasma}}{{L-Phenylalanine}_{\;plasma}}/\frac{{L-Valine}_{\;CSF}}{L{-Phenylalanine}_{\;CSF}}=R^{2}=0.67$). This means that 67% of the variance in y (the ratio between L-Valine/L-Phenylalanine in CSF) is explained by x (the ratio between L-Valine/L-Phenylalanine in plasma), which is an improvement compared to the single-metabolite correlations (Figure 3A–C). Sex differences in the correlations were minor for all ratio pairs (Table S2, Figure S2A,B). In particular, the amino acids showed a high correlation in their ratios.

**Figure 4:**
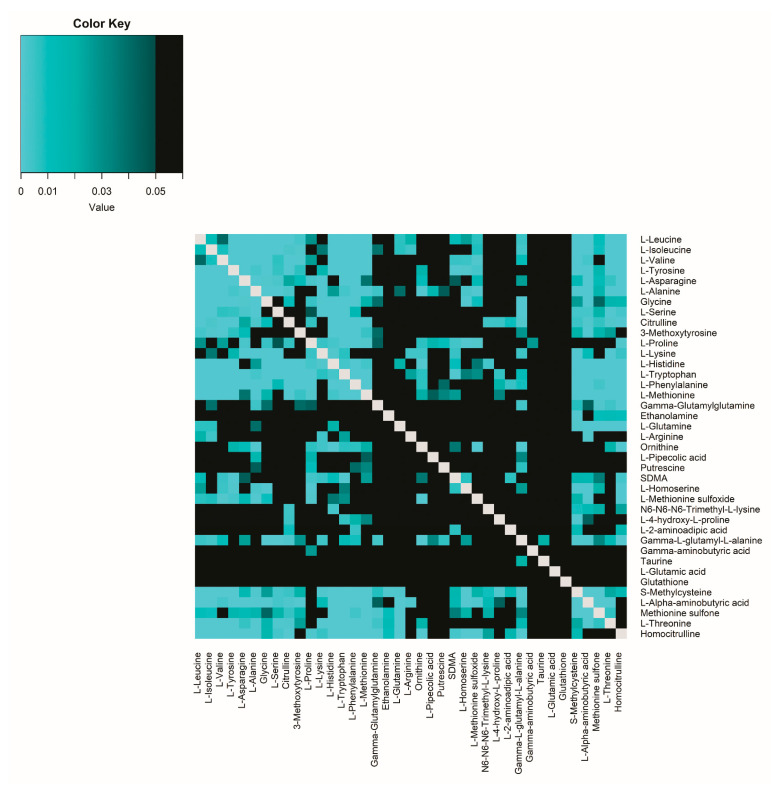
*p*-values of R^2^ gain. R^2^ in correlation ratios is significantly higher than the R^2^ of single correlations in healthy volunteers (FDR < 0.05). The lower the *p*-value value, the brighter blue the square; black squares are not significantly different (FDR < 0.05). Grey squares: *p*-value not applicable. R^2^ gain = the improvement of Plasma/CSF ratio correlations compared to single-metabolite ratio correlations.

The improvement in R^2^ ratio correlation (R^2^ gain) compared to single correlation is illustrated in Figure 4. The ratio correlations were significantly higher (after adjusting for multiple testing; FDR < 0.05) than the related single-metabolite correlations for 308 of 741 (41.5%) amine combinations (Figure 4, Table S3). As a control, correlations between the product of two amines were tested, but these showed no clear significance.

### 2.2. Amines in Plasma and CSF of Participants with Migraine

#### 2.2.1. Concentrations of Amines in Participants with Migraine

To apply our methodology to a pain disorder, we measured amine concentrations of plasma and CSF in 197 participants with migraine (*n* = 98 had migraine without aura (MO), *n* = 99 had migraine with aura (MA)) (for clinical characteristics see Table 4) (see Onderwater et al.’s paper [18] for details). For most amines, concentrations were higher in plasma than in CSF in participants with migraine without aura (Table 5) or migraine with aura (Table 6), similar to observations in healthy volunteers. Moreover, in migraine samples, ethanolamine and putrescine concentrations were higher in CSF than in plasma; L-Glutamine was the most abundant amine in CSF (migraine without aura: median: 589 mM and interquartile range: 516–637 mM; migraine with aura: median: 568 mM and interquartile range: 516–616 mM) with similar concentrations in blood (migraine without aura: median: 674 mM and interquartile range: 595–752 mM; migraine with aura: median: 653 mM and interquartile range 587–712 mM).

#### 2.2.2. Correlations Between Single-Metabolite Levels in Plasma and CSF in Participants with Migraine

As observed in healthy volunteers, in partipants with migraine most amines had a low correlation between plasma and CSF concentrations (Table 7 and Table S4). A total of 4 (homocitrulline, S-methylcysteine, methionine sulfone and L-alpha-aminobutyric acid) of the 39 amines (10.3%) had a correlation coefficient ≥ 0.70 in participants with migraine, both in migraine without aura and migraine with aura, similar to healthy volunteers. Overall, there were no clear differences between the correlations between the migraine subgroups (Table S4).

#### 2.2.3. Correlations Between Amine Metabolite Ratios in Plasma and CSF in Participants with Migraine

**Figure 5:**
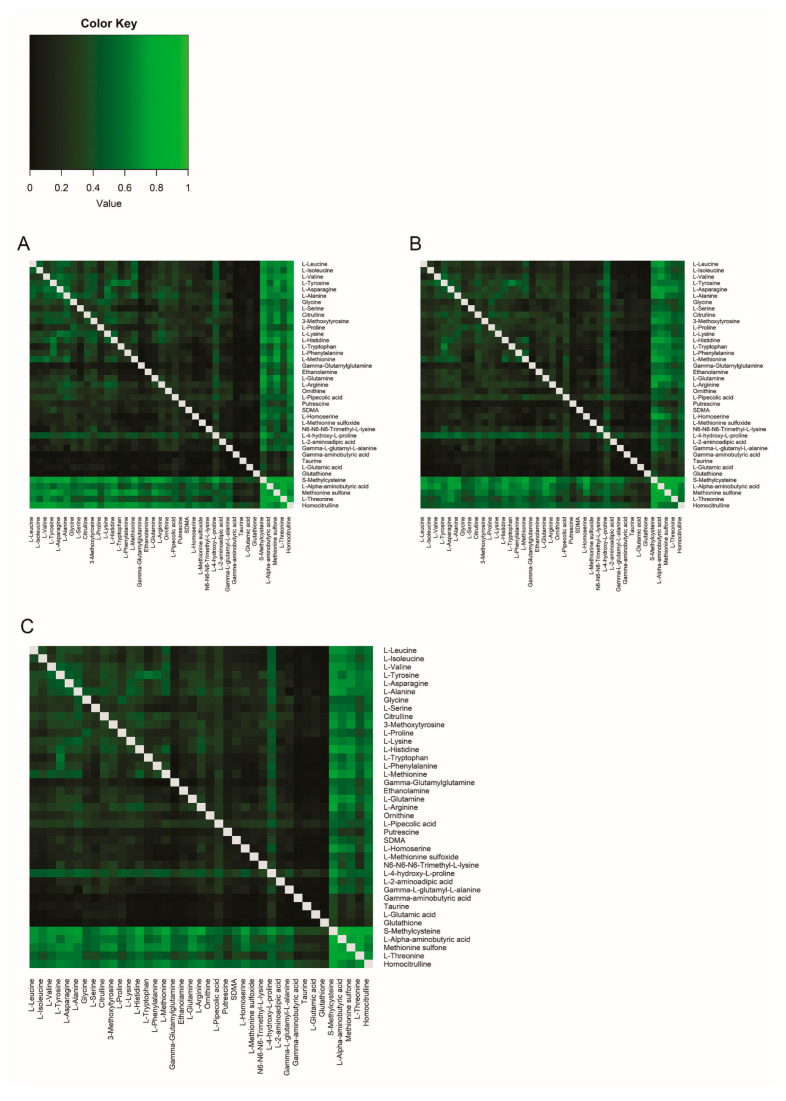
Correlation matrix of all possible metabolite ratios in participants with migraine. Coefficients of determination (R^2^) of all possible metabolite ratios are plotted in (**A**) migraine without aura participants (**B**) migraine with aura participants. (**C**) all participants with migraine together. The higher the correlation, the brighter the square.

For the participants with migraine the variance in y (the ratio between L-Valine/L-Phenylalanine in CSF) explained by x (the ratio between L-Valine/L-Phenylalanine in plasma) also clearly improves compared to single metabolite correlations (Figure 3D–F). Correlations between amine metabolite ratios in plasma and CSF in participants with migraine are presented in Figure 5 (see also Table S5). On visual inspection the results appear largely the same as in healthy volunteers, i.e., the correlation ratio of all amines, especially those ratios including S-methylcysteine, L-alpha-aminobutyric acid, methionine sulfone, L-threonine, and homocitrulline, remained high as was the case in healthy volunteers. However, a clear difference was observed when comparing the correlation matrix plot of healthy volunteers (Figure 2) with that of participants with migraine (Figure 5).

### 2.3. Comparison Between Healthy Volunteers and Participants with Migraine

#### 2.3.1. Comparison of Single-Metabolite Levels in Plasma and CSF in Healthy Volunteers and Participants with Migraine

Next, we compared the R^2^ single-metabolite level correlations of healthy volunteers with those of participants with migraine and found that there were no significant differences between healthy volunteers and participants with migraine (Table S6).

#### 2.3.2. Comparison of Correlations Between Amine Metabolite Ratios in Healthy Volunteers and Participants with Migraine

Finally, we compared the R^2^ correlations ratios of healthy volunteers with those of participants with migraine and found significant differences (after adjusting for multiple testing; FDR < 0.05) for the following combinations: L-Valine/L-Phenylalanine (FDR = 0.003), L-Arginine/S-Methylcysteine (FDR = 0.003), L-Alanine/L-4-hydroxy-L-proline (FDR = 0.015), L-Valine/L-4-hydroxy-L-proline (FDR = 0.025), L-Leucine/L-Phenylalanine (FDR = 0.028), and Gamma-L-glutamyl-L-alanine/L-4-hydroxy-L-proline (FDR = 0.041) (Table S7, Figure S3). Probably, the most relevant are L-Valine/L-Phenylalanine and L-Leucine/L-Phenylalanine, because the single-metabolite plasma/CSF correlation between healthy volunteers and participants with migraine did not differ significantly, whereas those of 4-hydroxy-proline and S-Methylcysteine appear to do so, although not significantly (Table S6). For L-Valine/L-Phenylalanine ratio and L-Leucine/L-Phenylalanine, the ratio correlation was lower in migraine participants compared to that in healthy volunteers (Table S7). This means that the difference in correlations between amine metabolite ratios between healthy volunteers and participants with migraine is likely not caused by the difference in one metabolite, but rather by the mechanism behind it.

## 3. Discussion

Given that many pain disorders are associated with metabolic changes in the CSF, it is vital for an accurate diagnosis, prognosis, and mechanistic understanding of the disorder to monitor those changes, preferably in the least burdensome manner for the patient, while still yielding useful information. Although CSF would be the preferred measurement, given its proximity to what happens in the brain, it is hardly a practical body fluid to obtain from patients when compared with measurements in blood, which can be drawn repeatedly from a patient. Thus, it is relevant to assess to what extent blood plasma levels of neurotransmitters and related amines correlate with those in CSF. To this end, we compared 39 amine concentrations in plasma and CSF, noticing that they generally did not correlate well. Hence, plasma concentrations per se are poor predictors of CSF concentrations for most of the amines. However, when studied as ratios, several amines correlated much better. One possible explanation for this finding is that ratios are more tightly regulated by the transport systems of the blood–brain barrier and probably reflect the co-transport of amines, and not so much the distribution of individual amines across the different body fluid compartments.

Only a limited number of studies have been conducted on the correlation between amine plasma and CSF concentrations. These studies mostly focused on proteinogenic amino acids and, like our results, did not yield clear correlations [14,15]. In our study, however, we also measured other amines and four of them showed strong single-metabolite correlation, namely homocitrulline, S-methylcysteine, methionine sulfone, and L-alpha-aminobutyric acid. Homocitrulline has been associated with energy metabolism and cerebellar dysfunction in animal studies [19,20] and S-methylcysteine may be neuroprotective after certain types of damage [21], but their exact biological roles in the brain are not fully known. For methionine sulfone and L-alpha-aminobutyric acid, no clear association with the brain is known. Most of these amines are involved in oxidation. However, much mechanistic insight regarding amines is still lacking; hence, no clear conclusion can be drawn from this.

Previous studies have not investigated whether there is a correlation between amine concentration ratios in plasma and CSF. In our study, the correlation of amine ratios was better than that of single-metabolite correlations, especially for amino acid pairs. Concentrations of nutrients and metabolites in the blood and the brain seem tightly regulated on both sides by the action of the blood–brain barrier. One consequence of this is that there is a concentration gradient between the brain and the rest of the body, with the concentration of amino acids in the brain typically being lower than in plasma [22,23]. This was consistent with our study, where we observed a lower concentration in CSF compared with plasma for most amines. Both the gradient and various transporters regulate the transport of amino acids across the blood–brain barrier [24,25,26,27]. The amino acids that showed a high gain in their correlation ratios compared to single-metabolite correlations in our study (L-leucine/L-methionine, L-leucine/L-asparagine, L-valine/L-phenylalanine) are all transported by the L amino acid transport system (LAT/L1), which is a sodium-independent transmembrane antiporter, consisting of two protein subunits, either a catalytic permease SLC7A5/LAT1 or SLC7A8/LAT2 and a regulatory glycoprotein (SLC3A2) [25,28,29]. Substrates transported by L1 include asparagine, glutamine, leucine, valine, methionine, histidine, isoleucine, tyrosine, tryptophan, phenylalanine, and threonine [25]. The L1 system is the main source through which essential neutral amino acids (NAAs) access the brain [25]. L1 imports large and neutral amino acids in exchange for intracellular amino acids [30]. Thus, the high correlations observed in our study may indicate tight regulation by this transporter, although we have no experimental data to prove that this is the case. Regardless, it is likely that the relative concentration, i.e., the abundance of an amino acid compared to a second amino acid, is important for transport. Classic neurotransmitters glutamic acid and GABA did not show strong correlations between plasma and CSF concentrations, either by themselves or as a ratio. Concentrations of amines related to the synthesis of neurotransmitters, such as glutamine and glutathione for glutamic acid and GABA and tryptophan for serotonin [5], also did not show a strong correlation between plasma and CSF. Thus, plasma levels of these metabolites do not seem to be useful to infer CSF levels from them.

As a secondary objective, we investigated whether the correlation of amine levels and/or ratios in CSF and blood of participants with migraine interictally differed compared to that in healthy volunteers. By comparing correlations between amine metabolite ratios between healthy volunteers and participants with migraine, six amine-related ratios (mainly amino acids) were found to be different, most likely due to an underlying dysfunction of the respective amine cotransporter, as four of the six changed ratios are related to the L1 transporter system [25] (Table S8). The ratio between amino acids is important for entry to the brain as there is competition between NAAs for this entry [31]. Additionally, the L-Arginine/S-Methylcysteine ratio was increased in migraine participants, while these amines are not transported by the L1 system. Arginine is transported by the y^+^ or cationic amino acid (CAA) transport (CAT) system [25,27]. The CAT system is primarily a CAA transporter, but also exhibits weak interactions with NAAs (phenylalanine, threonine, histidine, valine, methionine, serine, glutamine, alanine, and glycine) [25,32]. Our group previously found that arginine levels in CSF are reduced in migraine [18]. Arginine forms a caveolar complex with endothelial nitric oxide synthase (eNOS) to form nitric oxide (NO) [33]. NO has previously been implicated in migraine pathophysiology [34,35,36].

The exact mechanisms on how altered correlations between plasma and CSF ratios are related to migraine remains unclear, but this might be due to an alteration in the blood–brain barrier transport. However, an overall disruption cannot be concluded. Based on our disease model it can be concluded that studying ratios in the CNS, including in pain-related disorders, can provide useful insight into the pathophysiology of the disease. Although the current study does not allow for a detailed analysis of the transport mechanisms mentioned, the ratios may still be useful for assessing CNS metabolism of amines. For example, if a person has a high leucine/methionine ratio in plasma, this is probably also the case in the CSF. If this plasma ratio changes over time within this person, due to a particular disease process, one can envisage that the CSF ratio may also change. Of course, this needs to be proven in a longitudinal design with additional evidence that there is indeed a relationship between the specific ratio and the disease process. Although the current study is relevant for our understanding of disease mechanisms, the results cannot be directly transferred to clinical applications because the interpretation of ratios of amines, which in plasma and CSF seem to correlate better than the levels of individual amines, is at this moment rather difficult. This research mainly shows that when conducting research into blood-based biomarkers, one should consider whether this body fluid is representative of a pathological mechanism that occurs in the brain. Our data emphasizes that higher or lower levels of certain amines might not be the direct cause of migraine but rather a result of a possible underlying blood–brain barrier problem.

Strengths of this study include the large number of participants and the broad coverage of measured amine molecules. However, there are also limitations. The lack of intra-individually repeated measurements complicates validation of our findings and prohibits insight into dynamic changes of the measurements. To make predictions with ratios in healthy volunteers and CNS disorders, future (dynamic) studies should first of all replicate the identified correlations and ideally focus on obtaining longitudinal data with multiple (blood) measurements per individual at different time points (in relation to a provoked migraine attack as can be done with a migraine provoking agent).

## 4. Materials and Methods

### 4.1. Study Design

The primary aim of the study was to determine whether, which, and to what extent (1) single-amine metabolite concentration levels and (2) amine metabolite ratios based on these concentrations in the blood of healthy volunteers correlate with those in CSF. The secondary aim was whether such correlations were different in participants with migraine, as an example of a paroxysmal brain disease.

### 4.2. Study Population

Healthy volunteers and participants with migraine were recruited through a public advertisement or through the Leiden University Medical Center Headache Clinic. Additionally, participants (healthy volunteers and patients) were also recruited via the LUMINA (Leiden University Medical Centre Migraine Neuro Analysis) database. Blood and CSF were collected for research purposes from 96 healthy volunteers without migraine and 197 participants with migraine (*n* = 98 with aura and *n* = 99 without aura) outside attacks as part of a biochemical study on migraine pathophysiology [18]. To gain further insight into the pathophysiology of migraine, we here reinvestigated the data to assess whether the correlation of amine levels and/or ratios between CSF and blood were different in healthy volunteers and participants with migraine. For detailed information on the inclusion and exclusion criteria for both study populations, see Onderwater et al. [18]. In brief, the healthy volunteers had no signs or symptoms of a disease and no personal or family history of migraine or other regular headaches, nor did they have other neurological symptoms or other pain-related syndromes. They received reasonable financial compensation for undergoing a single lumbar puncture according to ethical guidelines approved by the LUMC Medical Ethics Committee. Migraine was diagnosed according to the International Classification of Headache Disorders [37]. The main exclusion criteria were severe neurological or severe psychiatric conditions, frequent headaches, oncological history, or contraindication for lumbar puncture (including signs and symptoms of increased intracranial pressure; local skin infection; or coagulopathy, including the use of anti-coagulant drugs or platelet inhibitors). Samples of participants with migraine were collected interictally and had to be free of an attack for at least three days prior to collecting body fluid.

### 4.3. Sample Collection

For details of CSF and blood sampling see Onderwater et al. [18]. For migraine participants, sample collection was performed interictally. In brief, CSF was collected between 2008 and 2016 by lumbar puncture before 1 pm and after an overnight fast (participants were allowed to drink only water 8 h before collection). Lumbar puncture was performed between the L3/L4, L4/L5, or L5/S1 interspace with a Quincke traumatic needle of 0.9 (20 G) × 90 mm (MediPlast, Malmö, Sweden) or an atraumatic Sprotte needle 0.9 (20 G), 0.7 (22 G), or 0.5 (24 G) × 90 mm (Pajunk, Geisingen, Germany). Intracranial pressure was measured, and 3 mL CSF was first collected for routine diagnostics (cell count, glucose and total protein level), before CSF was collected for metabolic measurements.

For metabolomics measurements, 3.8 mL CSF was sampled directly into a 15 mL polypropylene falcon tube (tube P1a; Cat. No. 188 271, Greiner Bio-One, Alphen aan de Rijn, The Netherlands) and centrifuged at 4 °C for 5 min (2000 rpm, 622 g) [38]. The supernatant was transferred into a new 15 mL polypropylene falcon tube, inverted several times, and divided into 0.5 mL aliquots into 1.8 mL Nunc^TM^ cryotubes (Cat. No. 368632, Sigma-Aldrich, Saint Louis, MO, USA) already containing 1.0 mL of cold ethanol (Prod. No. 8098, ethanol absolute, J.T.Baker, Phillipsburg, NJ, USA). The Nunc^TM^ cryotubes were inverted several times to thoroughly mix the CSF and ethanol and were placed on dry ice within 30 min of sampling. Aliquots were then transferred to −80 °C for storage within 60 min of sampling. Plasma samples were collected from the median cubital vein immediately after lumbar puncture; while participants rested in supine position, samples were drawn from the median cubital vein. Venous blood was collected in an EDTA plasma tube (Cat. No. 366643, BD Medical, Franklin Lakes, NJ, USA) and centrifuged for 20 min at 21 °C (2000 rpm, 622 g). The supernatant (plasma) was transferred into a new 15 mL polypropylene falcon tube, inverted several times, and divided into 0.5 mL aliquots in 1.0 mL Nunc^TM^ cryotubes (Cat. No. 366656, Sigma-Aldrich, Saint Louis, MO, USA). All aliquots were then transferred to −80 °C for storage within 60 min of sampling. All CSF and plasma samples remained at −80 °C until sample preparation.

### 4.4. Amine Measurements

CSF and plasma samples were measured using an ultra-performance liquid chromatography mass spectrometry (UPLC-MS) method that was shown to reliably quantify 74 biogenic amines for mouse CSF samples [16]. For details on sample preparation and amine measurements see Onderwater et al. [18]. In brief, quantitation of amino acids and biogenic amines utilized an AccQ-Tag derivatization strategy adapted from the protocol supplied by Waters (Milford, MA, USA). 5.0 µL of each sample was spiked with an internal standard solution. Proteins were precipitated by the addition of MeOH after which the samples were dried in a speedvac. The residue was reconstituted in a borate buffer (pH 8.8) with AQC reagent. LC–MS measurements were performed with an Acquity UPLC System (Waters) coupled to a QTRAP 6500 Triple-Quadruple MS System (Sciex, Framingham, MA, USA). For the chromatographic separation, 1 µL of the sample was injected on an AccQ-Tag Ultra 100 × 2.1 mm column with a particle size of 1.7 µm (Waters) and chromatographic separation was achieved with the flow rate of 0.7 mL/min over an 11 min LC gradient program.

The target analytes were detected in electrospray ionization (ESI) positive ion mode. The derivatized target metabolites and their internal standards were identified by their retention times and using their specific Multiple Reaction Monitoring (MRM) at nominal mass resolution. Data were pre-processed with MultiQuant Software for Quantitative Analysis v3.0.2 (Sciex, Framingham, MA, USA). Peak areas of target analytes relative to their corresponding internal standards were calculated as area ratios.

All samples were measured in ten separate batches including calibration (injected at the start and the end), QC, blank, and randomized study samples. Plasma samples were measured in the first five batches, whereas CSF samples were measured later. QC samples were analysed after every ten injections and used to monitor the intra-batch data quality and to correct for inter-batch analytical variance. To subtract the inter-batch analytical variance, all area ratios (study samples, calibration and blank samples) were corrected with a regression model generated through the data acquired from regular QC injections [39].

Concentration calculations were performed by using the Pearson’s linear correlation within the calibration range for both CSF and plasma samples. The R^2^ for each calibration line was selected ≥ 0.95. Based on corrected area ratios, the analytes were defined as not detected if 30% of the mean value of all study samples is equal to or less than the mean value of the lowest calibration level. The final concentration values were reported in µmol/L (µM) unit. Amines were only included when the inter-batch RSDqs was <15%. This was true for 47 out of 74 amines in CSF and 59 out of 74 amines in plasma. In addition, the interpolative calibration curve should be consistent between multiple batches. This was true for 42 out of 47 in CSF and 55 out of 59 in plasma.

Outlier detection was performed using PCA with a 99% confidence interval. Prior to the statistical analyses data were log_10_-transformed and values below [mean − 4 * SD] and above [mean + 4 * SD] were replaced with these cut-offs.

### 4.5. Statistical Analysis

#### 4.5.1. Correlations Between Single-Metabolite Levels in Plasma and CSF

To investigate whether amine levels in plasma and CSF were tightly linked and, consequently, whether the former are informative for the latter, we first calculated Pearson’s correlation coefficients (*r*) between plasma and CSF concentration for each amine separately. Metabolite levels were log_10_-transformed and adjusted for age, sex, and age*sex and were subsequently inverse normal transformed to obtain robust estimates of the correlation coefficients. Subsequently, we compared the plasma/CSF correlation coefficients between healthy subjects and subjects with migraine by normalizing *r* with Fisher’s transformation

$$z=0.5\frac{\ln\left(1+r\right)}{\ln\left(1-r\right)}$$

and subsequently testing the difference in *z*-values between groups against the centred normal distribution $N\left(0,\;\frac{1}{n_{1}-3}+\frac{1}{n_{2}-3}\right)$, with *n*_1_ and *n*_2_ being the sample sizes of groups.

#### 4.5.2. Correlations Between Amine Metabolite Ratios in Plasma and CSF

Since most plasma amine levels had only a weak correlation with CSF levels, we then investigated whether plasma amine ratios were correlated more strongly with the same amine ratio in CSF than could be expected based on the single amine correlations. For this, all 741 amine ratios were determined in plasma and CSF and correlation coefficients were calculated between them. Prior to the correlation analysis the ratios were log_10_-transformed, adjusted for age, sex, and age*sex, and then inverse normal transformed. We then tested for whether ratio correlation coefficients were higher than single metabolite correlations using the Steiger’s approach for comparing correlation coefficients in overlapping data [40]. Specifically, for a ratio of metabolites M1 and M2, we tested the difference between the Fisher-transformed correlation coefficient of the ratio $z_{M1/M2}$ and the average *z* of both metabolites $\frac{1}{2}z_{M1}+\frac{1}{2}z_{M2}$ one-sided against the centred normal distribution $N\left(0,\frac{1-(0.5s_{1}+0.5s_{2})}{n-3}\right)$, with *s_i_* the correction factor for overlapping data of M1/M2 relative to respectively M1 and M2, as defined in the Steiger’s approach [40]. Finally, we transformed the difference in *z*-value back with the inverse Fisher transformation to get a measure of the *gain* in the ratio correlation coefficient with respect to single metabolites.

$$r\;\mathrm{ratio}\;\mathrm{gain}\left(M1,M2\right)=\frac{\exp\left(2z_{M1/M2}-z_{M1}-z_{M2}\right)-1}{\exp\left(2z_{M1/M2}-z_{M1}-z_{M2}\right)+1}$$

Multiple test correction was performed for all statistical comparisons by using Benjamini Hochberg’s procedure. A *p* value < 0.05 was considered significant after false discovery rate (FDR) correction. Coefficients of determination (R^2^) and R^2^ ratio gains (R^2^ gain) for all metabolite ratios were plotted in a heatmap using R software (version 3.6.3 with package *gplots* and heatmap.2 function).

## 5. Conclusions

In conclusion, there is generally poor correlation between plasma and CSF concentrations for individual amine metabolites. However, ratios of certain amines show a good correlation between plasma and CSF. Some plasma amine measurements thus have the potential to be used to estimate CSF metabolite levels, which may be highly relevant for assessing fluctuations of amino acids and biogenic amines metabolism in CNS disorders, including pain-related disorders. As a proof of principle, we have shown that migraine shows changes in amine ratio correlations compared to healthy volunteers, indicating the possible involvement of specific blood–brain transporters. Hence, studying plasma ratios in pain-related disorders can provide useful insight into the pathophysiology of the disease and provide leads for future research.

## Figures and Tables

**Table 1:** Clinical characteristics of healthy volunteers.

	Healthy Volunteers
**Number of participants**	95
**Subject characteristics**	
Females (*n*, (%))	56 (58.9)
Years of age (±SD ^‡^)	38.8 (±14.5)
BMI ^†^ (±SD ^‡^)	23.7 (±2.8)
Smoking (*n*, (%))	20 (21.1)
**Overnight fasting**	
Fasting time in h (±SD ^‡^)	11.6 (±2.4)
**Sampling characteristics**	
Opening pressure in mmH_2_O (±SD ^‡^)	19.1 (±4.4)
**CSF characteristics**	
Erythrocytes count/3 µL (±SD ^‡^)	154 (±934)
Leukocytes count/3 µL (±SD ^‡^)	6 (±6)
Protein concentration in g/L (±SD ^‡^)	0.35 (±0.13)
Glucose in mmol/L (±SD ^‡^)	3.2 (±0.3)

^†^ BMI = body mass index; ^‡^ SD = standard deviation.

**Table 2:** Plasma and CSF concentrations in healthy volunteers.

Metabolite	Plasma (µM)			CSF ^†^ (µM)			Plasma/CSF Ratio
	M	Q1	Q3	M	Q1	Q3	M
Ethanolamine	12.088	11.004	13.338	22.679	20.239	24.404	0.55
Putrescine	0.105	0.083	0.13	0.161	0.123	0.198	0.67
L-Glutamine	665.43	600.158	714.841	591.26	538.944	637.634	1.12
N6-N6-N6-Trimethyl-L-lysine	0.468	0.39	0.568	0.404	0.365	0.447	1.17
Gamma-aminobutyric acid	0.146	0.123	0.169	0.099	0.071	0.121	1.51
L-Homoserine	0.134	0.12	0.15	0.06	0.053	0.066	2.25
Gamma-Glutamylglutamine	4.693	4.109	5.715	1.98	1.778	2.226	2.36
L-Arginine	78.796	66.678	94.937	24.216	21.507	26.295	3.4
L-Threonine	135.285	117.762	152.372	36.591	31.929	42.588	3.63
SDMA	0.61	0.549	0.68	0.163	0.139	0.199	3.65
L-Serine	108.619	92.113	121.252	28.161	25.711	30.606	3.78
L-Histidine	60.536	55.485	65.125	15.007	13.649	16.842	4.11
Homocitrulline	0.258	0.186	0.331	0.051	0.037	0.073	4.81
L-Phenylalanine	55.817	51.336	62.03	10.273	9.267	11.781	5.41
L-Alpha-aminobutyric acid	18.218	14.484	21.797	3.242	2.518	3.757	5.72
L-Methionine	23.506	21.018	26.282	3.904	3.544	4.455	5.8
L-Tyrosine	50.099	42.364	55.329	7.791	6.986	9.175	5.97
3-Methoxytyrosine	0.089	0.077	0.106	0.015	0.012	0.017	6.11
Methionine sulfone	0.285	0.242	0.376	0.047	0.037	0.063	6.17
L-Lysine	186.795	167.22	204.311	29.905	26.963	32.829	6.22
L-Asparagine	52.278	47.707	58.596	8.114	7.484	9.141	6.47
L-Methionine sulfoxide	0.736	0.648	0.828	0.099	0.082	0.114	7.49
S-Methylcysteine	3.64	3.106	4.86	0.461	0.369	0.622	7.92
L-2-aminoadipic acid	0.699	0.583	0.906	0.082	0.072	0.1	8.22
L-Leucine	277.057	244.39	318.336	33.685	29.966	39.018	8.45
L-Alanine	359.072	317.329	421.116	34.465	29.743	43.473	10.06
L-Isoleucine	116.684	103.751	132.595	11.702	10.009	13.24	10.46
Ornithine	65.305	55.212	74.791	5.359	4.799	6.309	11.6
L-Valine	242.729	212.533	277.108	20.728	17.63	23.222	11.88
L-4-hydroxy-L-proline	7.569	5.951	10.286	0.664	0.474	0.838	11.97
Gamma-L-glutamyl-L-alanine	0.507	0.411	0.61	0.041	0.036	0.046	12.01
Citrulline	29.169	25.484	33.029	2.168	1.836	2.638	12.93
Taurine	97.824	71.535	127.601	7.397	6.461	8.244	13.03
Glutathione	5.577	4.595	6.355	0.337	0.275	0.428	15.83
L-Tryptophan	56.353	49.209	63.568	2.385	2.146	2.703	23.28
Glycine	296.343	237.466	333.362	8.686	7.506	10.336	33.39
L-Pipecolic acid	11.26	9.687	13.694	0.34	0.258	0.41	34.28
L-Glutamic acid	44.036	34.697	63.195	0.399	0.35	0.447	110.32
L-Proline	178.184	140.29	218.478	0.922	0.751	1.233	183.31

Metabolites are ranked by plasma/CSF ratio from smallest to largest ratio. The table is based on uncorrected data. ^†^ CSF = cerebrospinal fluid. M = Median; Q1 = First quartile; Q3 = Third quartile.

**Table 3:** Correlations between single-metabolite levels in plasma and CSF in healthy volunteers.

Metabolite	*r*	R^2^	95% c.i.	FDR
Homocitrulline	**0.75**	0.56	0.41–0.68	6.16 × 10^−17^
S-Methylcysteine	**0.75**	0.56	0.41–0.68	6.16 × 10^−17^
Methionine sulfone	**0.75**	0.56	0.41–0.68	6.16 × 10^−17^
L-Alpha-aminobutyric acid	**0.73**	0.54	0.39–0.67	2.71 × 10^−16^
L-Threonine	0.69	0.48	0.32–0.62	5.03 × 10^−14^
3-Methoxytyrosine	0.64	0.40	0.24–0.55	3.11 × 10^−11^
L-Arginine	0.57	0.32	0.17–0.48	1.33 × 10^−8^
L-Glutamine	0.47	0.22	0.09–0.38	5.97 × 10^−6^
L-Lysine	0.46	0.21	0.08–0.37	1.11 × 10^−5^
L-Serine	0.46	0.21	0.08–0.37	1.20 × 10^−5^
L-Tyrosine	0.45	0.21	0.08–0.36	1.37 × 10^−5^
L-Proline	0.44	0.20	0.07–0.35	2.27 × 10^−5^
Citrulline	0.44	0.19	0.07–0.35	2.58 × 10^−5^
Glycine	0.43	0.18	0.06–0.34	4.56 × 10^−5^
L-Asparagine	0.42	0.17	0.05–0.33	6.85 × 10^−5^
Ethanolamine	0.41	0.17	0.05–0.32	9.12 × 10^−5^
L-Alanine	0.41	0.17	0.05–0.32	9.96 × 10^−5^
N6-N6-N6-Trimethyl-L-lysine	0.40	0.16	0.05–0.32	1.04 × 10^−4^
L-4-hydroxy-L-proline	0.36	0.13	0.03–0.28	6.05 × 10^−4^
L-Isoleucine	0.35	0.12	0.03–0.27	8.95 × 10^−4^
L-Leucine	0.35	0.12	0.02–0.26	1.12 × 10^−3^
Ornithine	0.34	0.12	0.02–0.26	1.29 × 10^−3^
L-Valine	0.33	0.11	0.02–0.25	1.70 × 10^−3^
Gamma-Glutamylglutamine	0.33	0.11	0.02–0.25	2.09 × 10^−3^
Putrescine	0.32	0.10	0.02–0.25	2.13 × 10^−3^
L-2-aminoadipic acid	0.30	0.09	0.01–0.23	4.59 × 10^−3^
L-Pipecolic acid	0.29	0.08	0.01–0.21	7.34 × 10^−3^
L-Histidine	0.25	0.06	0.00–0.18	2.31 × 10^−2^
Taurine	0.18	0.03	0.00–0.14	1.01 × 10^−1^
L-Tryptophan	0.17	0.03	0.00–0.13	1.38 × 10^−1^
L-Homoserine	0.16	0.03	0.00–0.13	1.40 × 10^−1^
L-Methionine	0.16	0.02	0.00–0.12	1.59 × 10^−1^
Gamma-L-glutamyl-L-alanine	0.15	0.02	0.00–0.12	1.64 × 10^−1^
SDMA	0.12	0.01	0.00–0.10	2.97 × 10^−1^
L-Glutamic acid	0.09	0.01	0.00–0.08	4.56 × 10^−1^
L-Phenylalanine	0.08	0.01	0.00–0.08	4.56 × 10^−1^
Gamma-aminobutyric acid	0.08	0.01	0.00–0.08	4.80 × 10^−1^
L-Methionine sulfoxide	0.06	0.00	0.00–0.07	5.58 × 10^−1^
Glutathione	0.02	0.00	0.00–0.05	8.63 × 10^−1^

Metabolites are ranked by value of this coefficient and values ≥ 0.7 are printed in bold. The colour gradient from blue to red depicts the strength of the correlation. The table is based on corrected data. *r* = Pearson correlation coefficient; R^2^ = coefficient of determination; 95% c.i. = 95% confidence interval of R^2^; FDR = false discovery rate.

**Table 4:** Clinical characteristics of participants with migraine.

	MO ^§^ Participants	MA ^¶^ Participants
**Number of participants**	98	99
**Subject characteristics**		
Females (*n*, (%))	60 (61.2)	66 (66.7)
Years of age (±SD ^‡^)	42.0 (±12.9)	41.7 (±13.5)
BMI ^†^ (±SD ^‡^)	23.6 (±2.5)	24.0 (±2.8)
Smoking (*n*, (%))	13 (13.3)	13 (13.1)
**Overnight fasting**		
Fasting time in hours (±SD ^‡^)	11.9 (±1.6)	11.7 (±1.6)
**Sampling characteristics**		
Opening pressure in mmH_2_O (±SD ^‡^)	18.0 (±4.7)	18.8 (±4.1)
**CSF characteristics**		
Erythrocytes count/3 µL (±SD ^‡^)	130 (±505)	2276 (±20,577)
Leukocytes count/3 µL (±SD ^‡^)	5.0 (±7)	21.0 (±89)
Protein concentration in g/L (±SD ^‡^)	0.35 (±0.10)	0.36 (±0.25)
Glucose in mmol/L (±SD ^‡^)	3.1 (±0.2)	3.2 (±0.3)

^†^ BMI = body mass index; ^‡^ SD = standard deviation; ^§^ MO = migraine without aura; ^¶^ MA = migraine without aura.

**Table 5:** Plasma and CSF concentrations in participants with migraine without aura.

Metabolite	Plasma (µM)			CSF ^†^ (µM)			Plasma/CSF Ratio
	Median	Q1	Q3	Median	Q1	Q3	Median
Ethanolamine	12.297	11.175	13.553	21.821	18.822	23.871	0.58
Putrescine	0.100	0.077	0.125	0.144	0.115	0.206	0.62
L-Glutamine	674.580	595.338	752.180	589.130	515.600	637.313	1.19
O-Phosphoethanolamine	10.010	6.639	13.745	7.752	6.437	8.995	1.22
N6-N6-N6-Trimethyl-L-lysine	0.513	0.418	0.610	0.384	0.343	0.454	1.28
Gamma-aminobutyric acid	0.141	0.127	0.163	0.100	0.074	0.129	1.41
L-Homoserine	0.132	0.119	0.144	0.058	0.053	0.064	2.31
Gamma-Glutamylglutamine	5.014	4.231	5.832	2.091	1.833	2.386	2.49
L-Arginine	80.671	60.536	94.243	23.307	20.515	26.424	3.50
L-Threonine	134.514	116.573	156.030	37.440	32.806	44.239	3.62
SDMA	0.600	0.540	0.680	0.152	0.131	0.190	3.79
L-Serine	104.086	95.945	119.437	27.274	24.831	31.276	3.95
L-Histidine	60.834	57.208	66.340	14.658	13.246	16.115	4.33
Homocitrulline	0.242	0.170	0.332	0.049	0.037	0.074	4.83
L-Phenylalanine	56.070	52.176	60.344	10.093	8.901	11.138	5.77
L-Alpha-aminobutyric acid	18.149	15.071	22.688	3.119	2.453	3.855	6.00
L-Tyrosine	49.079	43.117	55.010	8.053	6.849	9.219	6.20
L-Methionine	23.253	21.219	25.536	3.905	3.331	4.257	6.22
3-Methoxytyrosine	0.093	0.083	0.108	0.015	0.013	0.017	6.31
Methionine sulfone	0.304	0.220	0.381	0.045	0.035	0.060	6.37
L-Asparagine	51.826	46.566	57.312	7.849	6.839	9.082	6.66
L-Lysine	200.450	175.718	217.740	29.707	24.925	34.051	6.68
L-Methionine sulfoxide	0.723	0.619	0.806	0.097	0.081	0.117	7.22
S-Methylcysteine	3.744	3.037	4.854	0.464	0.371	0.647	8.14
L-Leucine	280.280	255.741	320.453	32.286	28.673	39.077	8.59
L-2-aminoadipic acid	0.775	0.648	0.997	0.092	0.080	0.104	8.61
L-Alanine	374.163	326.391	429.543	35.577	30.439	41.706	10.84
L-Isoleucine	118.753	104.690	133.144	11.132	9.200	13.814	11.03
Taurine	92.333	69.557	116.352	7.763	6.767	8.755	12.16
L-Valine	245.782	223.515	277.096	20.551	17.680	24.981	12.36
Gamma-L-glutamyl-L-alanine	0.532	0.441	0.672	0.044	0.040	0.051	12.60
L-4-hydroxy-L-proline	8.812	6.417	12.451	0.685	0.554	0.938	12.73
Ornithine	65.407	51.951	79.256	5.250	4.162	6.138	12.83
Citrulline	28.087	23.808	35.088	2.151	1.697	2.619	13.21
Glutathione	5.661	4.636	6.485	0.400	0.312	0.493	14.56
L.Tryptophan	57.474	51.837	61.938	2.284	2.000	2.700	24.75
Glycine	289.064	235.273	334.399	8.261	7.287	9.859	32.34
L-Pipecolic acid	10.778	8.670	14.545	0.323	0.257	0.403	35.21
L-Glutamic acid	40.028	32.485	54.316	0.387	0.355	0.478	107.70
L-Proline	171.004	140.167	209.404	0.984	0.805	1.235	181.27

Metabolites are ranked by plasma/CSF ratio from smallest to largest ratio. The table is based on uncorrected data. ^†^ CSF = cerebrospinal fluid. Q1 = First quartile; Q3 = Third quartile.

**Table 6:** Plasma and CSF concentrations in participants with migraine with aura.

Metabolite	Plasma (µM)			CSF ^†^ (µM)			Plasma/CSF Ratio
	Median	Q1	Q3	Median	Q1	Q3	Median
Ethanolamine	11.578	10.470	12.732	20.911	18.792	22.715	0.55
Putrescine	0.096	0.079	0.119	0.157	0.122	0.198	0.63
L-Glutamine	653.450	586.971	711.809	567.820	516.139	615.613	1.15
N6-N6-N6-Trimethyl-L-lysine	0.469	0.395	0.564	0.396	0.364	0.433	1.18
O-Phosphoethanolamine	9.092	6.046	13.232	6.789	6.028	7.943	1.24
Gamma-aminobutyric acid	0.141	0.126	0.162	0.098	0.073	0.123	1.46
L-Homoserine	0.132	0.117	0.147	0.060	0.053	0.065	2.28
Gamma-Glutamylglutamine	5.079	4.119	5.593	1.968	1.682	2.271	2.52
L-Arginine	72.078	60.850	81.213	22.499	19.179	24.445	3.22
SDMA	0.575	0.511	0.640	0.155	0.135	0.182	3.58
L-Threonine	131.388	114.403	146.662	37.090	30.923	41.640	3.61
L-Serine	109.030	93.263	124.880	28.032	24.813	32.959	3.86
L-Histidine	60.959	55.442	65.949	14.569	13.181	16.302	4.12
Homocitrulline	0.221	0.183	0.321	0.049	0.039	0.066	4.78
L-Phenylalanine	55.540	50.081	60.634	10.001	8.832	11.155	5.47
L-Alpha-aminobutyric acid	18.415	15.313	21.751	3.141	2.386	4.094	5.78
L-Tyrosine	49.443	42.673	56.030	8.247	6.846	9.526	5.81
L-Methionine	22.550	20.440	24.734	3.704	3.175	4.290	5.87
3-Methoxytyrosine	0.088	0.077	0.105	0.014	0.013	0.017	6.05
Methionine sulfone	0.290	0.230	0.363	0.048	0.036	0.061	6.21
L-Asparagine	49.761	46.959	54.779	7.571	6.818	8.638	6.57
L-Lysine	188.289	171.233	214.701	29.191	25.893	32.633	6.64
L-Methionine sulfoxide	0.730	0.624	0.828	0.098	0.079	0.120	7.51
S-Methylcysteine	3.529	2.837	4.965	0.491	0.364	0.640	7.56
L-2-aminoadipic acid	0.716	0.593	0.899	0.088	0.074	0.102	8.26
L-Leucine	264.337	240.759	307.927	32.152	27.056	37.816	8.44
L-Isoleucine	112.672	99.368	130.394	10.764	9.395	12.632	10.49
L-Alanine	379.229	326.339	442.442	34.173	30.171	40.508	10.74
Taurine	83.719	64.708	113.254	7.117	6.093	8.013	11.14
L-Valine	235.246	213.624	269.877	21.665	16.751	24.563	11.86
L-4-hydroxy-L-proline	7.770	6.020	10.363	0.644	0.485	0.832	12.10
Gamma-L-glutamyl-L-alanine	0.540	0.462	0.651	0.042	0.037	0.048	12.55
Citrulline	27.375	22.837	31.007	2.021	1.758	2.428	12.69
Ornithine	62.759	51.411	78.293	5.143	4.161	6.015	13.09
Glutathione	5.404	4.490	6.696	0.327	0.267	0.445	15.62
L-Tryptophan	54.546	47.679	59.223	2.338	2.098	2.608	23.17
Glycine	286.608	235.756	352.980	8.037	7.021	10.223	34.24
L-Pipecolic acid	10.626	8.529	12.854	0.313	0.258	0.397	34.61
L-Glutamic acid	39.946	28.893	58.014	0.391	0.359	0.458	99.13
L-Proline	168.383	133.030	213.782	0.910	0.749	1.177	174.50

Metabolites are ranked by plasma/CSF ratio from smallest to largest ratio. The table is based on uncorrected data. ^†^ CSF = cerebrospinal fluid. M = Median; Q1 = First quartile; Q3 = Third quartile.

**Table 7:** Correlations between single-metabolite levels in plasma and CSF in participants with migraine.

Metabolite	MO				MA			
	*r*	R^2^	95% c.i.	FDR	*r*	R^2^	95% c.i.	FDR
S-Methylcysteine	**0.84**	0.71	0.60–0.80	4.44 × 10^−26^	**0.86**	0.74	0.64–0.82	1.04 × 10^−28^
Methionine sulfone	**0.79**	0.63	0.50–0.74	2.94 × 10^−21^	**0.79**	0.62	0.49–0.73	3.25 × 10^−21^
Homocitrulline	**0.78**	0.61	0.47–0.72	2.86 × 10^−20^	**0.72**	0.51	0.36–0.64	8.16 × 10^−16^
L-Alpha-aminobutyric acid	**0.73**	0.54	0.39–0.66	9.66 × 10^−17^	**0.80**	0.63	0.50–0.74	1.43 × 10^−21^
L-Threonine	0.68	0.47	0.31–0.61	6.85 × 10^−14^	0.62	0.39	0.23–0.53	4.40 × 10^−11^
L-4-hydroxy-L-proline	0.63	0.40	0.24–0.55	1.86 × 10^−11^	0.64	0.41	0.26–0.56	6.02 × 10^−12^
L-Arginine	0.56	0.32	0.17–0.47	9.56 × 10^−9^	0.45	0.21	0.08–0.36	7.27 × 10^−6^
3-Methoxytyrosine	0.55	0.30	0.15–0.45	2.93 × 10^−8^	0.60	0.36	0.20–0.51	3.77 × 10^−10^
Citrulline	0.54	0.29	0.15–0.45	3.71 × 10^−8^	0.48	0.23	0.09–0.38	2.25 × 10^−6^
L-Alanine	0.54	0.29	0.14–0.45	3.71 × 10^−8^	0.44	0.19	0.07–0.34	1.84 × 10^−5^
L-Lysine	0.53	0.28	0.14–0.44	5.99 × 10^−8^	0.35	0.13	0.03–0.27	5.73 × 10^−4^
L-Pipecolic acid	0.52	0.27	0.12–0.42	1.57 × 10^−7^	0.56	0.32	0.17–0.47	6.38 × 10^−9^
L-Tyrosine	0.48	0.23	0.10–0.39	1.55 × 10^−6^	0.51	0.26	0.12–0.41	3.72 × 10^−7^
Ornithine	0.46	0.21	0.08–0.37	4.85 × 10^−6^	0.33	0.11	0.02–0.24	1.66 × 10^−3^
L-Asparagine	0.45	0.21	0.08–0.36	7.05 × 10^−6^	0.37	0.14	0.03–0.28	2.96 × 10^−4^
Glycine	0.41	0.17	0.05–0.32	7.11 × 10^−5^	0.36	0.13	0.03–0.27	4.58 × 10^−4^
L-Proline	0.38	0.15	0.04–0.29	2.27 × 10^−4^	0.47	0.22	0.09–0.38	2.78 × 10^−6^
L-Valine	0.37	0.14	0.03–0.28	3.74 × 10^−4^	0.38	0.14	0.04–0.29	2.23 × 10^−4^
N6-N6-N6-Trimethyl-L-lysine	0.36	0.13	0.03–0.28	5.13 × 10^−4^	0.43	0.18	0.06–0.34	2.53 × 10^−5^
L-Glutamine	0.35	0.12	0.02–0.26	9.34 × 10^−4^	0.40	0.16	0.05–0.31	8.91 × 10^−5^
L-Histidine	0.32	0.11	0.02–0.24	2.03 × 10^−3^	0.25	0.06	0.00–0.19	1.54 × 10^−2^
L-Isoleucine	0.32	0.10	0.02–0.24	2.04 × 10^−3^	0.22	0.05	0.00–0.16	4.06 × 10^−2^
Gamma-L-glutamyl-L-alanine	0.31	0.09	0.01–0.23	3.78 × 10^−3^	0.21	0.04	0.00–0.15	4.76 × 10^−2^
L-2-aminoadipic acid	0.30	0.09	0.01–0.22	4.49 × 10^−3^	0.41	0.16	0.05–0.31	8.31 × 10^−5^
Putrescine	0.29	0.08	0.01–0.21	6.09 × 10^−3^	0.19	0.04	0.00–0.14	7.30 × 10^−2^
L-Serine	0.28	0.08	0.01–0.21	7.59 × 10^−3^	0.40	0.16	0.05–0.31	8.41 × 10^−5^
L-Leucine	0.25	0.06	0.00–0.18	1.94 × 10^−2^	0.29	0.08	0.01–0.21	5.45 × 10^−3^
SDMA	0.23	0.05	0.00–0.17	2.88 × 10^−2^	0.10	0.01	0.00–0.09	3.79 × 10^−1^
L-Methionine	0.21	0.04	0.00–0.15	5.37 × 10^−2^	0.30	0.09	0.01–0.22	4.12 × 10^−3^
L-Methionine sulfoxide	0.20	0.04	0.00–0.15	6.20 × 10^−2^	0.19	0.04	0.00–0.14	6.49 × 10^−2^
Gamma-Glutamylglutamine	0.13	0.02	0.00–0.11	2.42 × 10^−1^	0.23	0.05	0.00–0.17	2.79 × 10^−2^
Ethanolamine	0.12	0.01	0.00–0.10	3.11 × 10^−1^	0.40	0.16	0.05–0.31	8.79 × 10^−5^
Gamma-aminobutyric acid	−0.11	0.01	0.00–0.09	3.29 × 10^−1^	0.04	0.00	0.00–0.06	7.21 × 10^−1^
Taurine	−0.11	0.01	0.00–0.09	3.35 × 10^−1^	0.20	0.04	0.00–0.15	6.29 × 10^−2^
L-Homoserine	0.11	0.01	0.00–0.09	3.37 × 10^−1^	0.32	0.10	0.02–0.24	2.19 × 10^−3^
L-Phenylalanine	−0.05	0.00	0.00–0.06	6.95 × 10^−1^	0.33	0.11	0.02–0.25	1.59 × 10^−3^
L-Glutamic acid	0.04	0.00	0.00–0.06	7.15 × 10^−1^	0.05	0.00	0.00–0.06	6.31 × 10^−1^
Glutathione	0.03	0.00	0.00–0.05	7.67 × 10^−1^	0.09	0.01	0.00–0.08	3.91 × 10^−1^
L-Tryptophan	−0.03	0.00	0.00–0.05	7.67 × 10^−1^	0.08	0.01	0.00–0.08	4.36 × 10^−1^

Metabolites are ranked by value of this coefficient and values ≥ 0.7 are printed in bold. The colour gradient from blue to red depicts the strength of the correlation. The table is based on corrected data. *r* = Pearson correlation coefficient; R^2^ = coefficient of determination; 95% c.i. = 95% confidence interval of R^2^; FDR = false discovery rate; MO = migraine with aura; MA = migraine without aura.

## Data Availability

Data not published within the article is available from the corresponding author upon reasonable request.

## Supplementary Materials

The following supporting information can be downloaded at: https://www.mdpi.com/article/10.3390/ijms26209899/s1.

## Abbreviations

The following abbreviations are used in this manuscript:

CSF	Cerebrospinal fluid
UPLC-MS	Ultra-performance liquid chromatography mass spectrometry
CNS	Central nervous system
GABA	γ-aminobutyric acid
QC	Quality control
PCA	Principal component analysis
MO	Migraine without aura
MA	Migraine with aura
ESI	Electrospray ionization
MRM	Multiple Reaction Monitoring
NAAs	Neutral amino acids
LAT/L1	L amino acid transport system
eNOS	Nitric oxide synthase
